# World TB Day — March 24, 2015

**Published:** 2015-03-20

Each year, World TB Day is observed on March 24. This annual event commemorates the date in 1882 when Robert Koch announced his discovery of *Mycobacterium tuberculosis*, the bacterium that causes tuberculosis (TB). World TB Day provides an opportunity to raise awareness about TB-related problems and solutions and to support worldwide TB control efforts.

For the second year, CDC supports the theme “Find TB. Treat TB. Working together to eliminate TB.” Health officials in local and state TB programs are encouraged to provide educational awareness regarding TB to their communities and to work with other agencies and organizations that care for those most at risk for TB.

In 2014, a total of 9,412 new cases of TB were reported in the United States, a rate of 3.0 per 100,000 population (1). Although the total number of TB cases continues to decline, 2014 showed the smallest decline in incidence in over a decade. Nationally, TB still persists at greater incidence in foreign-born persons and racial or ethnic minorities.

CDC is committed to a world free from TB. Initiatives to improve awareness, testing, and treatment of latent TB infection and TB disease among groups at high risk are critical to achieve elimination of TB in the United States.

Additional information regarding World TB Day and CDC’s TB elimination activities is available at http://www.cdc.gov/tb/events/worldtbday.
